# Effect of Topically Applied Anaesthetic Formulation on the Sensitivity of Scoop Dehorning Wounds in Calves

**DOI:** 10.1371/journal.pone.0163181

**Published:** 2016-09-20

**Authors:** Dominique McCarthy, Peter Andrew Windsor, Charissa Harris, Sabrina Lomax, Peter John White

**Affiliations:** School of Life and Environmental Sciences, Faculty of Veterinary Science, University of Sydney, Sydney, NSW, Australia; University of Bari, ITALY

## Abstract

The post-operative effects of three formulations of topical anaesthetic and a cornual nerve block on the sensitivity of scoop dehorning wounds in calves were compared in two trials. In Trial 1, 21 female Holstein dairy calves aged 8 to 24 weeks were randomly allocated to two groups: (1) scoop dehorning with a post-operative application of a novel topical anaesthetic powder (DTAP, n = 10); and (2) scoop dehorning with a post-operative application of a novel topical anaesthetic ethanol liquid (DTAE, n = 11). In Trial 2, 18 castrated male and 18 female Hereford beef calves aged 16 to 20 weeks were randomly allocated to four groups: (1) scoop dehorning with a pre-operative cornual nerve block of lignocaine (DCB, n = 9); (2) scoop dehorning with a post-operative application of the novel topical anaesthetic ethanol liquid from Trial 1 (DTAE, n = 9); (3) scoop dehorning with a post-operative application of a topical anaesthetic gel (DTAG, n = 9); and (4) sham dehorning (CON, n = 9). Sensitivity was assessed by scoring the behavioural response of calves to stimulation of the wound or skin at time points before and after treatment. In Trial 1, DTAP calves had a greater probability of displaying more severe responses than DTAE calves at 90 and 180 min (*P* < 0.001). In Trial 2, at 1 h, DTAG calves had a greater probability of displaying more severe responses than CON calves. At 2 h onwards, all dehorned calves had a greater probability of displaying more severe responses than CON calves (*P* < 0.001). There were no differences between the responses of DCB, DTAG and DTAE calves at any time point. Topical anaesthetic formulations result in almost immediate but temporary anaesthesia of the wound following scoop dehorning in calves and may provide a practical option for pain relief on-farm.

## Introduction

Dehorning of cattle is painful, yet remains a commonly performed procedure in horned breeds [1] as it reduces injuries from social contact, improves safety for stock-persons [2], dehorned cattle require less space during feeding and transport [3] and dehorning reduces bruise trim at slaughter by approximately 50% [4]. As bruising of cattle costs the Australian beef industry approximately $30 million annually [5], retention of horns has major economic implications.

The northern beef industry in Australia is very extensive, with average property size reported at 23,436 ha for 2014–2015 [6]. The majority of cattle breeds in this region are *Bos Indicus* or *B*. *indicus* crosses and most calves are born with horns. Animal management on these properties differs greatly from the smaller southern Australian properties and from that of most other beef producing nations in that cattle are handled infrequently during the year. The age at which beef calves are ‘marked’ (that is, animals are mustered and subjected to husbandry management procedures including ear tagging, branding, vaccination and dehorning) varies, but is usually in excess of 3.5 months of age [1]. The horn tissue at this stage is more developed than in dairy calves that are usually dehorned within 1 to 3 months of birth. In northern Australia, most beef calves are dehorned by horn amputation, using scoop, cup or knife dehorning tools [7]. This method of dehorning involves removal of the horn as well as a small area of surrounding skin [8] and has been found to cause significant pain and distress [9–11].

Numerous studies have shown that a cornual nerve block using local anaesthetics (LA) such as lignocaine is effective at alleviating the intra-operative and acute post-operative pain caused by dehorning [10–13]. However, the impracticality of injectable drug administration in this environment has prevented widespread adoption of this technique by Australian producers [1].

Topically applied LA may provide an alternative option to address the post-operative pain of dehorning wounds as administration does not involve extra handling time or a high level of skill. Previous studies have demonstrated the efficacy of a topical anaesthetic formulation (Tri-Solfen®, Bayer Animal Health, Pymble NSW Australia) for use during mulesing [14], tail docking and surgical castration [15] in lambs and surgical castration in beef calves [16]. Preliminary studies have shown that modified formulations of Tri-Solfen® applied to dairy calves during scoop dehorning significantly reduced the sensitivity of wounds up to 1.5 h [17] and 5 h [18] following the procedure.

The overall aim of the previous studies [17, 18] and the current study, are to investigate options for providing pain relief to calves undergoing scoop dehorning that are both effective and practical to use on-farm.

## Materials and Methods

### Animals and Housing

Experimental protocols were approved by the Animal Ethics Committee of The University of Sydney (Approval No. 5832). The study involved two trials on separate groups of calves to investigate the effect of three different topical anaesthetic products on wound sensitivity post dehorning. In trial 1, calves were sourced from a commercial dairy, “Schofields”, in the Southern Highlands, NSW, Australia. In trial 2, calves were sourced from a commercial beef herd, “Ayrston”, in the Central Tablelands of NSW, Australia. At the conclusion of the trials, calves remained on the properties and continued to be used as commercial livestock.

#### Trial 1

Twenty-one female horned Holstein-Friesian dairy calves aged 8 to 24 weeks undergoing routine dehorning were used in this trial. Prior to experimentation, calves were moved from group pens into a holding pen adjacent to the cattle handling facilities, where they remained for the duration of the trial. The calves in this trial had been separated from their mothers at birth and hand reared as per routine dairy farm practice.

#### Trial 2

Eighteen castrated male and 18 female unweaned horned Hereford beef calves aged 16 to 20 weeks undergoing routine dehorning were used in this trial. Calves were moved from the paddock into a holding pen adjacent to the cattle handling facilities 2 days before the trial, where they were habituated to movement through handling facilities twice daily for 2 days before experimentation. Other than during time when moved through the handling facilities, the calves had access to their dams.

#### Topical anaesthetic products

Tri-Solfen® is a registered and commercially available local anaesthetic and antiseptic gel for topical application immediately post mulesing of merino lambs. It contains 40.6 g/L lignocaine, 4.2 g/L bupivacaine, 5 g/L cetrimide and 24.8 mg/L adrenalin. We have been studying the efficacy of this product for pain relief during a number of surgical interventions in farm animals [19], including various modifications of the original formula to enhance adherence and efficacy during scoop dehorning [17, 18].

Trial 1 compared the practicality and efficacy of two novel topical anaesthetic agents developed for dehorning (Bayer Animal Health, Pymble NSW Australia). Both formulations contained 20% w/v lignocaine and 4% w/v bupivacaine. These were specifically designed for application to amputation dehorning wounds where haemorrhage may affect absorption of anaesthetic agents. Higher concentrations of lignocaine and bupivacaine were included in these novel formulations with the intention of increasing the amount of active ingredients coming into contact with the tissue surface immediately upon application. The first formulation used an inert powder base as a carrier in an attempt to improve adherence to the wound. The second formulation used an ethanol / water base as a carrier designed to evaporate following application. The most effective formulation from this trial was used in the second trial comparing the practicality and efficacy with that of Tri-Solfen® and a cornual nerve block (2% w/v lignocaine).

In both trials, practicality was assessed through observations of product application and efficacy was evaluated through assessment of skin and wound sensitivity.

### Experimental Design and Treatments

#### Trial 1

The trial was conducted over 1 day. On the day of the trial, calves were moved one at a time through the race and restrained in a head bale (Australian Stockyard Co, Goulburn NSW Australia) for treatment and data collection. Calves were blocked by age and randomly allocated to one of two treatments by use of computer generated random numbers (Microsoft Excel 2007, Microsoft Corporation): (1) scoop dehorning with a post-operative application of a novel topical anaesthetic powder (Bayer Animal Health, Pymble NSW Australia) (DTAP, n = 10); and (2) scoop dehorning with a post-operative application of a novel topical anaesthetic ethanol liquid (Bayer Animal Health, Pymble NSW Australia) (DTAE, n = 11). Approximately 5 to 10 g of powder was applied to DTAP calf wounds with a measuring spoon and approximately 4 mL of the ethanol liquid was applied to DTAE calf wounds using a household spray bottle. The amount of ethanol liquid sprayed from the bottle was calibrated using a 3 mL syringe. The liquid was sprayed into a cup and the syringe used to measure the liquid and it was determined there was 2 mL of product released per spray. The products were applied immediately after dehorning so as to completely cover the wound and cut skin edge. Dehorning was performed by a single, experienced technician using a medium size scoop dehorning device (Bainbridge Barnes Dehorner, The Farm Store, Melbourne, VIC, Australia). Dehorning was performed by placing the dehorner over the horn and pulling apart the handles to excise the horn and surrounding skin. The procedure of dehorning and applying the ethanol spray or the powder took approximately 15 s or 30 s per animal, for each of these products respectively. Data was collected immediately prior (0 h) to treatment, then 1 min, 90 min and 180 min post treatment. Between data collections, calves were released into a holding yard.

#### Trial 2

The trial was conducted over 2 days. On each day of the trial, 18 calves were moved one at a time through the race and restrained in a head bale (Australian Stockyard Co, Goulburn NSW Australia) for treatment and data collection. Calves were blocked by age, sex and day of trial and randomly allocated to one of four treatments by use of computer generated random numbers (Microsoft Excel 2007, Microsoft Corporation): (1) scoop dehorning with a pre-operative cornual nerve block of lignocaine (Ilium Lignocaine 20®, Troy Laboratories, Glendenning NSW Australia) (DCB, n = 9); (2) scoop dehorning with a post-operative application of the ethanol liquid from Trial 1 (Bayer Animal Health, Pymble NSW Australia) (DTAE, n = 9); (3) scoop dehorning with a post-operative application of a topical anaesthetic gel (Tri-Solfen®, Bayer Animal Health, Pymble NSW Australia) (DTAG, n = 9) and (4) sham dehorning (CON, n = 9). Calves in the DCB group were administered a cornual nerve block 15 min prior to dehorning. An 18 G needle was inserted to a depth of 1 cm immediately behind the temporal ridge at a point midway between the lateral canthus of the eye and the base of the horn [20, 21]. Lignocaine (5 mL) was injected into the tissue in the vicinity of the cornual nerve of each horn and anaesthesia of the horn area was confirmed by the pinprick test with an 18 G needle immediately prior to dehorning. For calves in the DTAE and DTAG groups, approximately 4 mL of product was applied to each wound immediately after dehorning so as to completely cover the wound and cut skin edge. Dehorning was performed as described for Trial 1. The ethanol spray and the gel were applied using household spray bottles and the amount applied per spray was calibrated as described for Trial 1. The procedure of dehorning and applying the ethanol liquid or the gel took approximately 15 s per animal. Sham dehorning was performed by placing the dehorner over the horn bud and applying light pressure to the surrounding skin, without excising any tissue. Data was collected immediately prior (0 h) to treatment, then 1 h, 2 h, 4 h and 6 h post treatment. Between data collections, calves were released into a holding yard.

### Assessment of Skin and Wound Sensitivity

**Fig 1:**
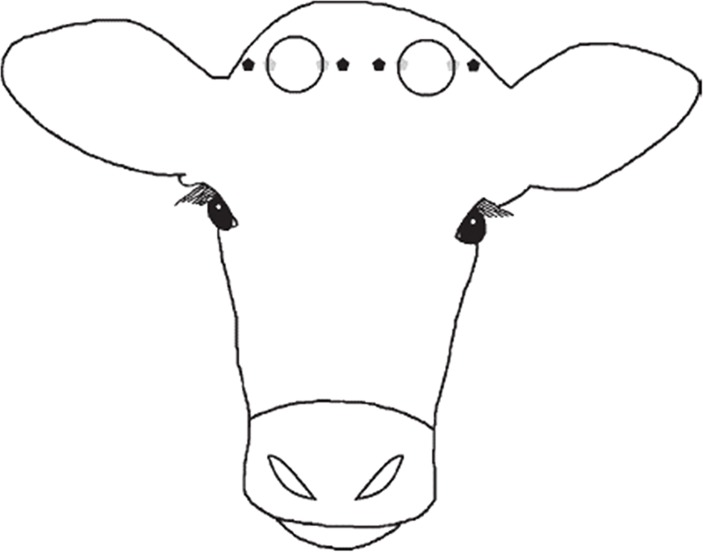
Sites Subjected to Sensory Testing. Grey stars represent sites on the edge of the horn base or wound (Area 1) and black stars represent sites on the skin surrounding the horn or wound (Area 2).

Nociceptive and anti-nociceptive responses were noted in both trials. Mechanical stimulation of the horn or wound was performed using von Frey monofilaments (Touch-Test® Sensory Evaluators, North Coast Medical and Rehabilitation Products, CA USA). This was performed at two sites on the immediate edge of the horn base or wound (Area 1) and two sites on the skin surrounding the horn or wound (Area 2) (Fig 1). Area 2 sites were 2 cm from the edge of the horn base or wound. Von Frey monofilaments are calibrated to bend at a pre-determined pressure. A 75 g/f (light touch) and 300 g/f (pain) monofilament were used to determine allodynia and hyperalgesia, respectively. Side (left or right horn) and site were randomised for each repeated measure. Calves were blindfolded during measurement to eliminate visual stimuli and reduce stress and consequent struggling behaviours. Sensitivity was assessed by scoring the behavioural responses of the calves to mechanical stimulation on a numerical rating scale of 0 to 3 adapted from Espinoza *et al*. [17] whereby: 0 = no response; 1 = mild response including minor withdrawal reflex such as a slight head movement or an ear flick; 2 = moderate response including partial withdrawal reflex such as partial head rotation; and 3 = severe response including full withdrawal reflex such as full head jerk or rotation.

### Statistical Analysis

Sample size calculation was based on the following assumptions: the cornual nerve block and sham treatments will have response scores of 0 or 1, where the topical anaesthetic groups will have median scores 1 or 2, with an assumed standard deviation (SD) of 1.8. Using the equation (a + b)^2^ x 2(SD)^2^/(mean1-mean2)^2^ and assuming a Type 1 error (a) of 5% and a Type 2 error (b) of 80%, 8.13 animals per group would be required. Therefore a minimum of 9 animals per group were included to allow for error in estimates of the means and SD. All data was analysed using ordinal logistic regression (OLR) in ASReml® 3.0 statistical software (VSN International Ltd, Hemel Hempstead UK). For Trials 1 and 2, the fixed effects of the OLR model were Treatment x Time, Area and von Frey. In Trial 1, Calf was included as a random effect. In Trial 2, Calf, Day of trial, Age and Sex were included as random effects. Data is presented as cumulative odds ratios with the statistical probabilities of calves in each treatment group displaying response score *Y* = 0, 1, 2 and 3. For all statistical calculations, *P* values ≤ 0.05 were considered statistically significant.

## Results

There were no adverse clinical effects registered for any of the animals following treatment.

### Trial 1

**Fig 2:**
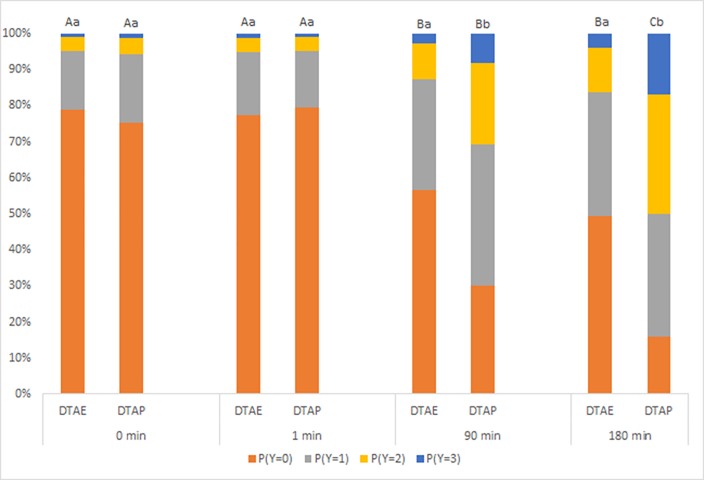
Probability of calves from Trial 1 in each treatment group displaying responses (Y; 0 = no response, 1 = mild, 2 = moderate, 3 = severe) at different time points. Results combine the effect of both von Frey monofilaments and all sites tested. (DTAE = scoop dehorned and treatment with topical anaesthetic ethanol spray; and DTAP = scoop dehorned and treatment with topical anaesthetic powder). a-b Within each time point, treatment groups not sharing a common letter are significantly different (*P* < 0.05). A-C Within each treatment, time points not sharing a common letter are significantly different (*P* < 0.05).

There was a significant Time x Treatment interaction (*P* < 0.001) (Fig 2).

All calves had a greater probability of displaying a more severe response at 90 min than at 1 min. DTAP calves also had a greater probability of displaying a more severe response at 180 min than at 90 min. DTAP calves were more likely to display more severe responses than DTAE calves at 90 and 180 min.

**Fig 3:**
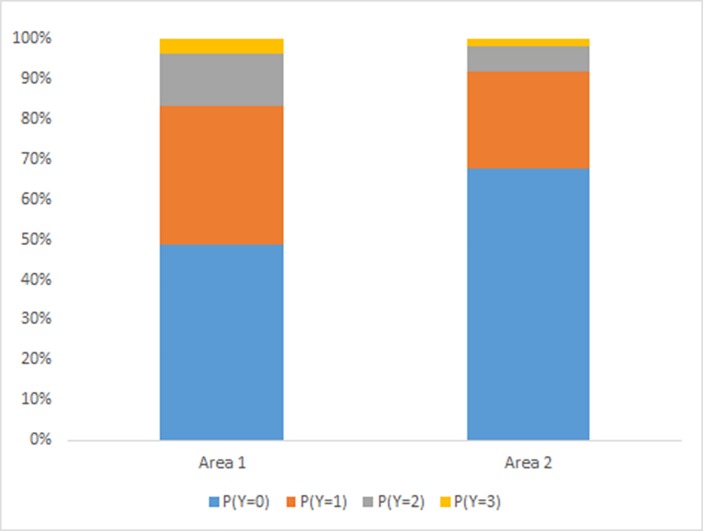
Probability of calves from Trial 1 displaying responses (Y; 0 = no response, 1 = mild, 2 = moderate, 3 = severe) to stimulation of different Areas. Results combine the effect of both von Frey monofilaments, both treatment groups and all time points.

There was a significant effect of Area (*P* < 0.001) (Fig 3). Calves had a greater probability of displaying more severe responses for Area 1 than for Area 2.

There was no significant effect of von Frey (*P* = 0.916).

Application of the ethanol liquid via a spray bottle was easier and quicker than application of the powder via a spoon, with less wastage of product. The powder was difficult to apply directly to the dehorned area and it was noted that wastage was an issue, particularly in windy conditions, raising health and safety concerns for the operator with potential inhalation of the powder during application.

### Trial 2

**Fig 4:**
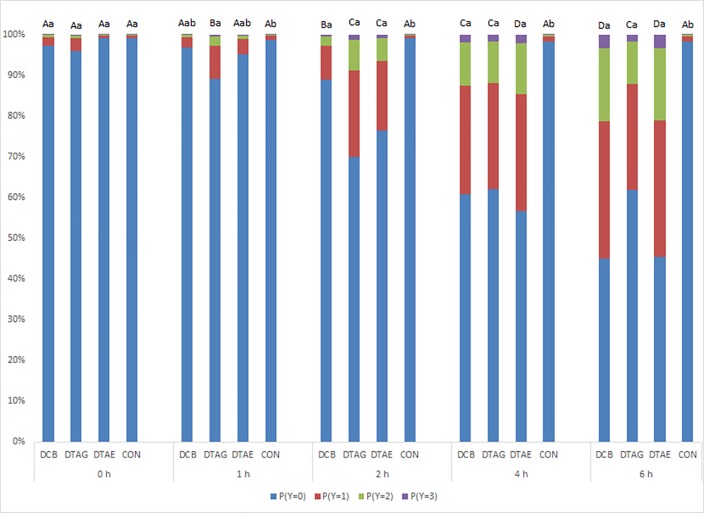
Probability of calves from Trial 2 in each treatment group displaying responses (*Y*; 0 = no response, 1 = mild, 2 = moderate, 3 = severe) at different time points. Results combine the effect of both von Frey monofilaments and all sites tested. (DCB = scoop dehorned and treatment with cornual nerve block; DTAG = scoop dehorned and treatment with a topical anaesthetic gel; DTAE = scoop dehorned and treatment with a topical anesthetic ethanol spray; and CON = sham dehorned). a–c Within each time point, treatment groups not sharing a common letter are significantly different (*P* < 0.05). A-D Within each treatment, time points not sharing a common letter are significantly different (*P* < 0.05).

There was a significant Time x Treatment interaction (P<0.001) (Fig 4). DCB calves had an increasing probability of displaying more severe responses at each time point from 1 h onwards. DTAG calves had an increasing probability of displaying more severe responses at each time point from 0 to 2 h. DTAE calves had an increasing probability of displaying more severe responses at each time point from 0 to 4 h. Prior to treatment (0 h), there were no differences between any treatment groups. At 1 h, DTAG calves were more likely to display a more severe response than CON calves. From 2 h onwards, all dehorned calves were more likely to display more severe responses than CON calves.

**Fig 5:**
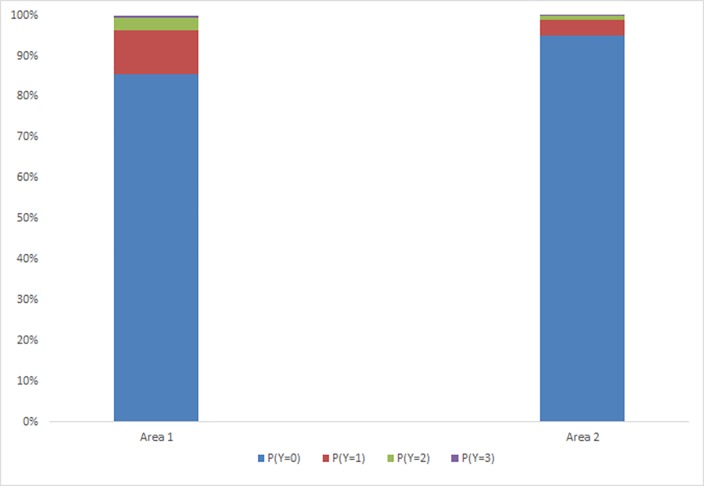
Probability of calves from Trial 2 displaying responses (Y; 0 = no response, 1 = mild, 2 = moderate, 3 = severe) to stimulation of different Areas. Results combine the effect of both von Frey monofilaments, all treatment groups and all time points.

There was a significant effect of Area (*P* < 0.001) (Fig 5). Calves had a greater probability of displaying more severe responses for Area 1 than for Area 2.

**Fig 6:**
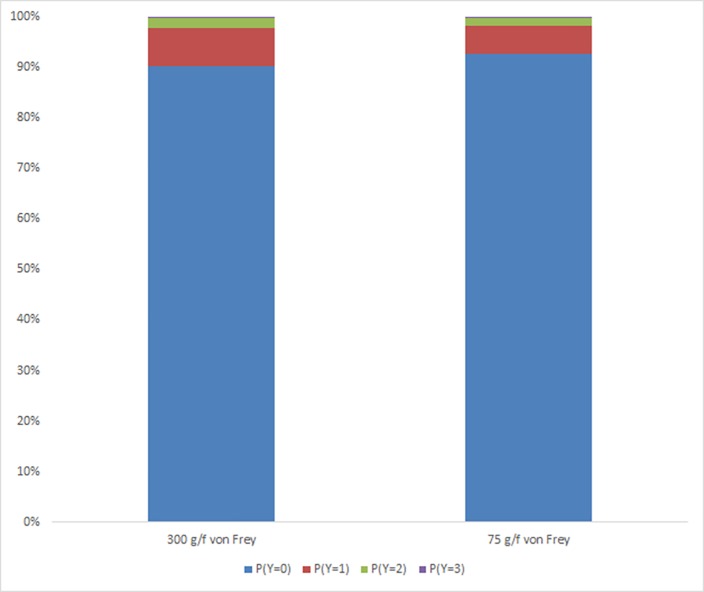
Probability of calves from Trial 2 displaying responses (Y; 0 = no response, 1 = mild, 2 = moderate, 3 = severe) to stimulation with different von Frey monofilaments. Results combine the effect of both Areas, all treatment groups and all time points.

There was a significant effect of von Frey (*P* = 0.007) (Fig 6). Calves had a greater probability of displaying more severe responses to the 300 g/f von Frey than to the 75 g/f von Frey.

Administration of the cornual nerve block took more time and required more skill than application of the ethanol liquid and the gel via a spray bottle.

## Discussion

Amputation dehorning is widely used in the Australian beef industry, causing an open wound and haemorrhage, plus pain and distress to calves [1, 8]. Currently, there are no commercially available, farmer-applied anaesthetic or analgesic options for pain management for dehorning in Australia. While a cornual nerve block has been shown to effectively minimise acute pain associated with the procedure [9, 10], it has limitations for use in an extensive setting [1]. The current study extended previous studies on the efficacy of modified formulations of Tri-Solfen® for dehorning in calves [17, 18], comparing the effects of three formulations of topical anaesthetic and a cornual nerve block on wound sensitivity in scoop dehorned calves.

Firstly, the effects of two novel topical anaesthetics designed for use on scoop dehorning wounds were compared, to assess which formulation was more effective at providing wound anaesthesia. The ease of application was also observed to evaluate the practicality of these products for use in a farm setting. These novel formulations were designed to improve absorption of anaesthetic agents in the presence of arterial haemorrhage resulting from the scoop dehorning procedure [8]. This issue was identified in a previous study investigating the use of the topical anaesthetic gel for surgical tail-docking wounds in lambs [15]. In this study it was noted that arterial bleeding associated with surgical tail removal may have prevented effective adherence of the product to the wound, resulting in reduced efficacy [15]. Although modified formulations of Tri-Solfen® have shown some efficacy for scoop dehorning of cattle [17, 18], compromised adherence of a gel product to the wound may still be an issue [17]. Hence the investigation of the powder and ethanol / water base carriers of topical anaesthetic in this study.

Trial 1 demonstrated that the ethanol spray was more effective than the powder, as shown by greater anti-nociceptive responses to wound stimulation at 90 and 180 min post treatment. Responses of all calves increased in severity from 1 to 90 min after dehorning, indicating heightened sensitivity of the wound. Wound sensitivity continued to increase in DTAP calves from 90 to 180 min, suggesting a waning effect of the powder after 90 min compared to the ethanol spray.

The efficacy and practicality of the ethanol spray, a topical anaesthetic gel and a cornual nerve block were then compared in Trial 2. Sham dehorned calves had the greatest probability of anti-nociceptive responses (score 0) to stimulation at all time points, indicating an absence of pain or hypersensitivity in the intact tissue. The increase in response severity seen in all dehorned calves over time demonstrated a hyperalgesic progression associated with the pain escalation response of skin incisions or open wounds [14, 22]. There was no change in sensitivity from before treatment to 1 h post treatment in DCB calves, suggesting effective local anaesthesia. From 2 h onwards, response severity of DCB calves increased at each time point, indicating diminishing efficacy. Similar responses have been reported in previous work investigating the efficacy of a lignocaine cornual nerve block on cortisol [10] and behavioural responses [9] of dehorned calves. The response score severity of DTAG and DTAE calves increased up to 2 and 4 h, respectively, however did not change up to 6 h. This suggests a delayed anaesthetic effect for the spray-on formulations which persisted longer than the cornual nerve block. The ethanol spray contained a much higher concentration of anaesthetic agents compared to the other treatments. This could explain the extended duration of anaesthesia compared to the cornual nerve block. In addition, topical application of the anaesthetic agents may have impacted the rate of absorption, as suggested in previous studies [16, 23, 24]. Extended duration of topical lignocaine applied to burn wounds in humans has been reported [23]. It was suggested that the gradual absorption of the lignocaine from a cream base resulted in extended anaesthesia. Prolonged efficacy of the topical anaesthetic gel formulation up to 24 h post treatment has been observed in mulesed sheep [24] and castrated calves [16]. The vasoconstrictive properties of adrenaline in the formulation may contribute to slowing the rate of systemic absorption of the anaesthetic agents, therefore concentration at the wound site is protracted. Additionally, the effect of a wound barrier created by the gel base is suggested to attenuate pain, possibly by covering damaged nerve endings and protecting the wound from exposure to the environment and stimulation [24]. Extension of the observation period beyond 6 h should be considered in future studies on the duration of efficacy of topical anaesthetic for dehorning wounds.

There were no treatment differences in responses of DCB, DTAG or DTAE calves at any time point, suggesting that the post-operative efficacy of all anaesthetic treatments was similar up to 6 h. DCB and DTAE calves responded similarly to CON calves at 1 h post treatment, indicating effective local anaesthesia at this time. DTAG calves tended to have more mild and moderate responses compared to CON calves at this time point which again could be attributed to a slower rate of absorption, as mentioned previously [16, 24].

There were very few severe response scores displayed by any dehorned calves at all time points, particularly at 1 h and 2 h post treatment (Fig 4), suggesting an anaesthetic effect. Alternatively this could indicate that the 300 g/f was not eliciting a noxious pain stimulus, resulting in less severe responses. In Trial 2, although there was a greater probability of calves having more severe responses to stimulation with the 300 g/f von Frey than the 75 g/f, this was only marginal (Fig 6).

Conclusions from this study are limited by the lack of a comparison to an untreated dehorned group of calves, omitted due to welfare concerns for such animals. However, the comparable results of the post-operative spray-on formulations to the injected LA in the cornual nerve block demonstrates efficacy of these products [9, 10, 12].

Von Frey stimulation of both Area 1 and 2 produced nociceptive responses from calves in both Trials 1 and 2. The effect of Area is a reflection of primary and secondary hyperalgesia, with stimulation of Area 1 (wound site) eliciting greater severity of response from calves than that of Area 2 (uncut surrounding tissue) within the 180 min (Trial 1) and 6 h (Trial 2) observation periods. Primary hyperalgesia develops at the site of injury due to sensitised nociceptors. Secondary hyperalgesia develops in the tissue surrounding the site of injury and is due to central sensitisation [25]. Secondary hyperalgesia is a consequence of primary hyperalgesia and therefore tends to develop at a slower rate [16] when accumulation of inflammatory mediators, initiated from wound injury, results in depolarisation of terminal nerve endings and excitation of nociceptors. The inflammatory mediators take time to accumulate and a pain response is only initiated when an excitation threshold is reached, resulting in gradual or delayed development of secondary hyperalgesia [26].

This is the first time that these current formulations of topical anaesthetic have been investigated for use on wounds of dehorned calves and compared to a cornual nerve block for post-operative pain relief. The results of this study warrant further investigation into the pain relieving effects of topical anaesthetic for calves undergoing scoop dehorning as its post-operative ease of administration was superior to a cornual nerve block and apparent efficacy in desensitising dehorning wounds was comparable to that of a lignocaine cornual nerve block.

## Supporting Information

S1 File Experimental data set. (XLSX)

## References

[1] Petherick JC. Animal welfare issues associated with extensive livestock production: The northern australian beef cattle industry. Appl Anim Behav Sci. 2005;92: 211–234.

[2] Kupczynski R, Budny A, Spitalniak K, Tracz E. Dehorning of calves—methods of pain and stress alleviation—a review. Ann Anim Sci. 2014;14: 231–243.

[3] Stafford KJ, Mellor DJ. Dehorning and disbudding distress and its alleviation in calves. Vet J. 2005;169: 337–349. 15848777 10.1016/j.tvjl.2004.02.005

[4] Prayaga KC. Genetic options to replace dehorning in beef cattle—a review. Aust J Agric Res. 2007;58: 1–8.

[5] CSIRO. Breeding hornless cattle [Internet]. Commonwealth Scientific and Industrial Research Organisation (CSIRO); 2015 [updated 27 February 2015; cited 2016 10 February]. Available: http://www.csiro.au/en/Research/AF/Areas/Animal-Science/Premium-livestock-breeds/Hornless-Cattle. Accessed 10 February 2016.

[6] Martin P. Australian Beef: financial performance of beef cattle producing farms, 2012–13 to 2014–15 Canberra, Australia: Australian Bureau of Agricultural and Resource Economics and Sciences; 2015 14.7.

[7] Irwin J, Walker B. Dehorning Cattle [Internet]. NSW Department of Primary Industries; 1998 [updated 1 September 1998; cited 2016 26 April]. Available: http://www.dpi.nsw.gov.au/agriculture/livestock/beef/husbandry/general/dehorning-cattle. Accessed 26 April 2016.

[8] Stafford KJ, Mellor DJ. Painful husbandry procedures in livestock and poultry In: Grandin T, editor. Improving animal welfare: a practical approach. Wallingford, UK: CABI; 2009 p. 88–114.

[9] Sylvester SP, Stafford KJ, Mellor DJ, Bruce RA, Ward RN. Behavioural responses of calves to amputation dehorning with and without local anaesthesia. Aust Vet J. 2004;82: 697–700. 15977616 10.1111/j.1751-0813.2004.tb12162.x

[10] Sylvester SP, Mellor DJ, Stafford KJ, Bruce RA, Ward RN. Acute cortisol responses of calves to scoop dehorning using local anaesthesia and/or cautery of the wound. Aust Vet J. 1998;76: 118–122. 9578782 10.1111/j.1751-0813.1998.tb14542.x

[11] Sutherland MA, Mellor DJ, Stafford KJ, Gregory NG, Bruce RA, Ward RN. Cortisol responses to dehorning of calves given a 5-h local anaesthetic regimen plus phenylbutazone, ketoprofen or adrenocorticotropic hormone prior to dehorning. Res Vet Sci. 2002;73: 115–123. 12204628 10.1016/s0034-5288(02)00005-x

[12] Petrie NJ, Mellor DJ, Stafford KJ, Bruce RA, Ward RN. Cortisol responses of calves to two methods of disbudding used with or without local anaesthetic. N Z Vet J. 1996;44: 4–8. 16031884 10.1080/00480169.1996.35924

[13] McMeekan CM, Mellor DJ, Stafford KJ, Bruce RA, Ward RN, Gregory NG. Effects of local anaesthesia of 4 to 8 hours' duration on the acute cortisol response to scoop dehorning in calves. Aust Vet J. 1998;76: 281–285. 9612552 10.1111/j.1751-0813.1998.tb10160.x

[14] Lomax S, Sheil M, Windsor PA. Impact of topical anaesthesia on pain alleviation and wound healing in lambs after mulesing. Aust Vet J. 2008;86: 159–168. 10.1111/j.1751-0813.2008.00285.x 18454833

[15] Lomax S, Dickson H, Sheil M, Windsor PA. Topical anaesthesia alleviates short-term pain of castration and tail docking in lambs. Aust Vet J. 2010;88: 67–74. 10.1111/j.1751-0813.2009.00546.x 20402687

[16] Lomax S, Windsor PA. Topical anesthesia mitigates the pain of castration in beef calves. J Anim Sci. 2014;91: 4945–4952. 10.2527/jas.2012-5984 23965386

[17] Espinoza C, Lomax S, Windsor P. The effect of a topical anesthetic on the sensitivity of calf dehorning wounds. J Dairy Sci. 2013;96: 2894–2902. 10.3168/jds.2012-5954 23477817

[18] Espinoza CA, McCarthy D, White PJ, Windsor PA, Lomax SH. Evaluating the efficacy of a topical anaesthetic formulation and ketoprofen, alone and in combination, on the pain sensitivity of dehorning wounds in Holstein-Friesian calves. Anim Prod Sci. 2015.

[19] Windsor PA, Lomax S, White PJ. Progress in pain management to improve small ruminant farm welfare. Small Rumin Res. 2016: In press.

[20] Edmondson MA. Local and regional anesthetic techniques In: Lin H, Walz P, editors. Farm animal anesthesia: cattle, small ruminants, camelids and pigs. Chichester, UK: John Wiley & Sons, Inc.; 2014 p. 136–154.

[21] Clarke KW, Trim CM. Anaesthesia of cattle. Veterinary Anaesthesia 11 ed. Edinburgh, New York: Saunders/Elsevier, 2014; 2014. p. 313–340.

[22] Redua MA, Valadao CAA, Duque JC, Balestrero LT. The pre-emptive effect of epidural ketamine on wound sensitivity in horses tested by using von Frey filaments. Vet Anaesth Analg. 2002;29: 200–206. 28404363 10.1046/j.1467-2995.2002.00083.x

[23] Brofeldt BT, Cornwell P, Doherty D, Batra K, Gunther RA. Topical lidocaine in the treatment of partial-thickness burns. J Burn Care Rehabil. 1989;10: 63–68. 2921260 10.1097/00004630-198901000-00009

[24] Lomax S, Sheil M, Windsor PA. Duration of action of a topical anaesthetic formulation for pain management of mulesing in sheep. Aust Vet J. 2013;91: 160–167. 10.1111/avj.12031 23521101

[25] Meyer RA, Ringcamp M, Campbell JN, Raja SN. Neural Mechanisms of hyperalgesia after tissue injury. Johns Hopkins APL Tech Dig. 2005;26: 56–66.

[26] Gregory NG. Pain Physiology and behaviour of animal suffering. Oxford, UK: Blackwell Science; 2004.

